# Low Molecular Weight, 4-*O*-Sulfation, and Sulfation at Meta-Fucose Positively Promote the Activities of Sea Cucumber Fucoidans on Improving Insulin Resistance in HFD-Fed Mice

**DOI:** 10.3390/md20010037

**Published:** 2021-12-29

**Authors:** Shiwei Hu, Sichun Chen, Hongli Zhu, Mengyu Du, Wei Jiang, Yu Liu, Xiang Gao, Laijin Su, Yangli Xu

**Affiliations:** 1 National Engineering Research Center for Maine Aquaculture, Zhejiang Ocean University, Zhoushan 316022, China; hushiweihai@163.com (S.H.); cscpurity@163.com (S.C.); zhuhongli2020@163.com (H.Z.); dumengyu816@163.com (M.D.); jiangwei_zjou@163.com (W.J.); liuyu1987@zjou.edu.cn (Y.L.); 2 College of Life Sciences, Qingdao University, Qingdao 266071, China; 3 Anqiu Huatao Food Co., Ltd., Weifang 262100, China; 4 College of Life and Environmental Science, Wenzhou University, Wenzhou 325035, China; 5 Wenzhou Characteristic Food Resources Engineering and Technology Research Center, Wenzhou Academy of Agricultural Science, Wenzhou 325006, China; xuyangli@163.com

**Keywords:** sea cucumber, fucoidans, low molecular weight, 4-*O*-sulfation, sulfation at ortho-fucose, insulin resistance

## Abstract

Fucoidans from sea cucumber (SC-FUC) have been proven to alleviate insulin resistance in several species. However, there are few studies that clarify the relationship between their structure and bioactivity. The present study evaluated the influence of molecular weight (Mw), sulfation concentrations (Cs), and sulfation position on improving insulin resistance using SC-FUC. Results showed that fucoidans with lower Mw exerted stronger effects. Having a similar Mw, *Acaudina molpadioides* fucoidans (*Am*-FUC) with lower Cs and *Holothuria tubulosa* fucoidans with higher Cs showed similar activities. However, *Isostichopus badionotus* fucoidans (higher Cs) activity was superior to that of low-Mw *Thelenota ananas* fucoidans (*Ta*-LFUC, lower Cs). Eliminating the effects of Mw and Cs, the bioactivity of *Am*-FUC with sulfation at meta-fucose exceeded that of *Ta*-FUC with sulfation at ortho-position. Moreover, the effects of *Pearsonothuria graeffei* fucoidans with 4-*O*-sulfation were superior to those of *Am*-LFUC with 2-*O*-sulfation. These data indicate that low Mw, 4-*O*-sulfation, and sulfation at meta-fucose contributed considerably to insulin resistance alleviation by SC-FUC, which could accelerate the development of SC-FUC as a potential food supplement to alleviate insulin resistance.

## 1. Introduction

The occurrence and development of insulin resistance is a complex pathological process caused by a decline in insulin sensibility of insulin target organs, subsequent blocking of insulin signaling transduction, and an ultimate decrease in intracellular glucose uptake and utilization [1]. In insulin resistance, insulin is unable to effectively combine with insulin receptors, and subsequently, the phosphoinositide 3-kinase/protein kinase B signaling is blocked [2]. This eventually leads to significant decreases both in both glucose transporter 4 (Glut4) translocation from the cytoplasm to the cytomembrane and glycogen synthase phosphorylation (p-GS) [3,4]. On the other hand, there is a decline in the activities of hexokinase (HK) and pyruvate kinase (PK), which are crucial for glucose utilization in cells to produce energy or glycogen synthesis [5]. Moreover, the activities of glycogen phosphorylase (GP) and glucose-6-phosphatase (G6Pase) are elevated, which catalyze gluconeogenesis reactions to release glucose in the blood [6]. Increasing attention has been paid to insulin resistance because of its adverse effects. It has been confirmed that insulin resistance underlies the morbidity of several metabolic disorders, including obesity, inflammation, type 2 diabetes mellitus, and certain cancers [7,8,9]. There were more than 650 million obese people in the world in 2019, of which over 80% showed significant insulin resistance and over 420 million suffered from diabetes [10]. According to the International Diabetes Federation, approximately 90% of type 2 diabetic patients exhibit apparent insulin resistance, and the number of the patients is likely to exceed 600 million by 2035 [11]. Thus, prevention and control of insulin resistance have been an important goal in human health.

Fucoidans from sea cucumber (SC-FUC) are polysaccharides comprised of an L-fucose backbone and a sulfate group backbone [12]. SC-FUC are widely considered to possess great potential as a functional food ingredient profiting due to their various bioactivities, including anti-oxidant, anti-tumor, anti-hyperglycemia, hematopoiesis, and anti-hyperlipidemia activities, among others [13,14,15]. 

It has been widely acknowledged that the structural features of polysaccharides determine their biological activities. Chain conformation, molecular weight (M_w_), and sulfation are considered important factors that significantly influence fucoidans derived from marine invertebrates [16]. Linear structures with uniform repeating units are the universal in SC-FUC, which has been identified as ideal for investigating their structure–function relationship [17]. SC-FUC have uniform repeating tetrasaccharide units with an α-1,3-glycosidic linkage, with their differences lying in their molecular weight, sulfation concentrations (C_s_), sulfation pattern (2-*O*-, 4-*O*-, or 2,4-*O*-sulfo group substitution), and sulfation location (sulfo group at ortho-fucose or meta-fucose) depending on the species. Several SC-FUC have been demonstrated to improve insulin resistance, including fucoidans from *Acaudina molpadioides* (*Am*-FUC) [18], *Isostichopus badionotus* (*Ib*-FUC) [19], *Cucumaria frondosa* [20], *Holothuria leucospilota* [21], and fucoidans from *Pearsonothuria graeffei* (*Pg*-FUC) [22]. However, the effect of the structure–function relationship of SC-FUC on insulin resistance is yet to be determined.

**Figure 1:**
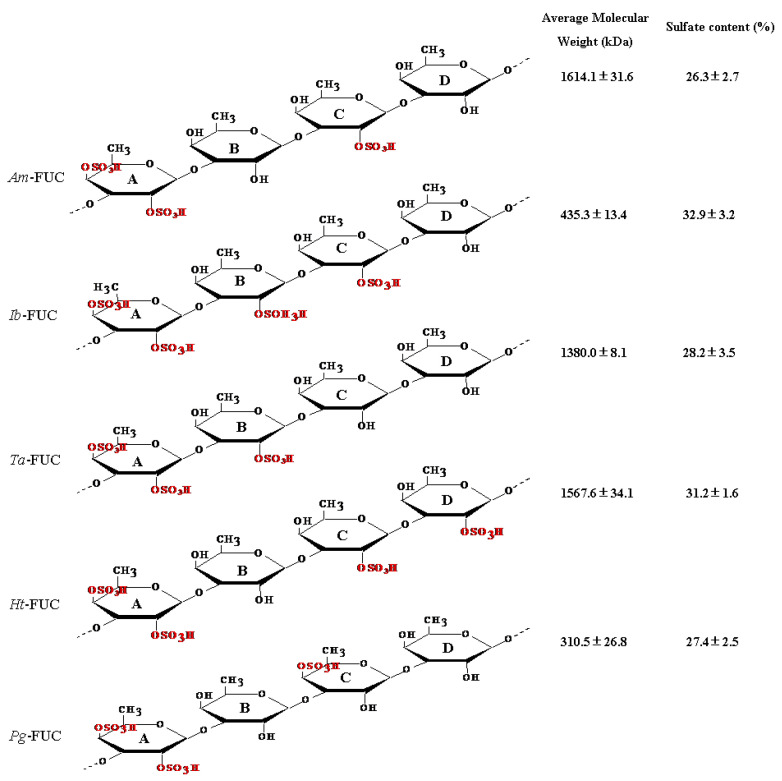
The structure, molecular weight, and sulfation concentration of fucoidans from 5 sea cucumbers. 2,4DS-fucose was defined as fucose A, following with fucoses B, C, and D. Sulfation at fucose B and D (neighboring fucose A) was named sulfation at ortho-fucose position, while sulfation at fucose C (spacing fucose A) was named sulfation at meta-fucose position [19,20]. *Acaudina molpadioides* fucoidans, *Am*-FUC; *Isostichopus badionotus* fucoidans, *Ib*-LFUC; *Thelenota ananas* fucoidans, *Ta*-LFUC; *Holothuria tubulosa* fucoidans, *Ht*-LFUC; *Pearsonothuria graeffei* fucoidans, *Pg*-LFUC.

In the present study, we aimed to investigate the effect of the structure–function relationship of SC-FUC on alleviating insulin resistance in high-fat diet (HFD)-induced insulin-resistant mice. For M_w_, *Am*-FUC with different M_w_ by an intracellular enzyme (*Wenyingzhuangia fucanilytica* CZ1127) were used to explore insulin resistance mitigation effects [23]. To determine the influence of C_s_, *Am*-FUC and *Ht*-FUC, depolymerized *Ta*-FUC and *Ib*-FUC were used to comparatively analyze the activities of SC-FUC with similar M_w_ but different C_s_. *Am*-FUC (sulfo group at meta-fucose) and *Ta*-FUC (sulfo group at ortho-fucose) with similar M_w_ and C_s_ were comparatively examined for the influence of structure–function sulfation location. To determine the influence of structure–function sulfation pattern, depolymerized *Am*-LUC (with a 2-*O*-sulfation) and *Pg*-FUC (with a 4-*O*-sulfation) were comparatively analyzed (Figure 1).

## 2. Results

### 2.1. Effects of Fucoidans with Different M_w_ on Insulin Resistance

**Figure 2:**
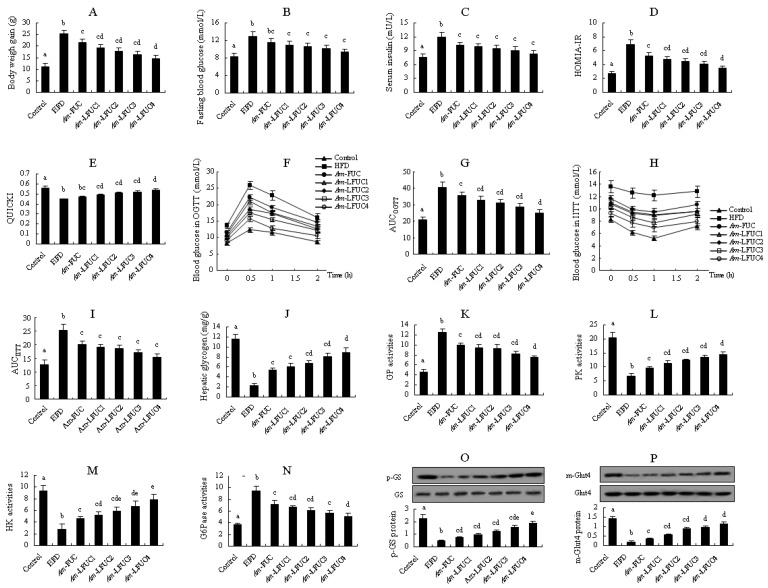
Effects of *Am*-FUC with different molecular weights on alleviating insulin resistance in HFD-fed mice. *Acaudina molpadioides* fucoidans, *Am*-FUC. *Am*-FUC and the 4 depolymerized *Am*-LFUCs had the similar sulfate contents and different Mw. (**A**), Body weight gain; (**B**), Fasting blood glucose; (**C**), Serum insulin; (**D**), homeostasis model assessment of insulin resistance index (HOMA-IR) value; (**E**), quantitative insulin sensitivity check index (QUICKI) score; (**F**), oral glucose tolerance test (OGTT); (**G**), areas under the curve (AUC) in OGTT test; (**H**), intraperitoneal insulin tolerance test (IITT); (**I**), AUC in IITT test; (**J**), Hepatic glycogen; (**K**), GP activities; (**L**), PK activities; (**M**), HK activities; (**N**), G6Pase activities; (**O**), p-GS protein expression (p-GS and GS, 85 kDa); (**P**), m-Glut4 protein expression (m-Glut4 and Glut4, 43 kDa). Body weight gain was assessed using 12 mice. OGTT and IITT were assessed using 6 mice, respectively. p-GS protein was assessed using the 3 mice with 40 U/kg insulin stimulation while m-Glut4 protein was assessed with 0.5 U/kg insulin. The other parameters were assessed using the 6 mice treated with normal saline. Different lowercase letters represent significant difference (*p* < 0.05) compared between groups.

We evaluated the insulin resistance-alleviation activities of fucoidans with different M_w_ using *Am*-FUC and the four depolymerized *Am*-LFUCs. As shown in Figure 2A, *Am*-FUC and the four *Am*-LFUCs all caused significant decreases in body weight gain (*p* < 0.05), and the effect of *Am*-LFUC4 was better than that of *Am*-FUC (*p* < 0.05). Notably, the smaller the fucoidans M_w_, the lower the body weight gain observed in experimental mice compared with the HFD group.

Blood glucose and insulin levels, including HOMIA-IR and QUICKI values, blood glucose changes in OGTT and IITT experiments, are usually the common parameters to indicate insulin resistance. There was no significant difference in fasting blood glucose between the HFD and *Am*-FUC groups. However, fasting blood glucose levels were remarkably reduced by 15.13, 17.38, 21.02, and 27.31% in the four *Am*-LFUCs groups compared with the HFD group (*p* < 0.05 Figure 2B). For serum insulin concentration, the *Am*-FUC and the four *Am*-LFUC groups significantly lowered the serum insulin level by 14.57, 18.07, 21.15, 24.83, and 30.89% (*p* < 0.05 Figure 2C) compared with the HFD mice, respectively. HOMIA-IR values were significantly decreased in *Am*-FUC and the four *Am*-LFUC groups (*p* < 0.05 Figure 2D) compared with the HFD mice, and the activity of *Am*-LFUC4 was superior to that of *Am*-FUC (*p* < 0.05). QUICKI scores were remarkably increased in the four *Am*-LFUC groups (*p* < 0.05), and *Am*-LFUC4 was more effective than *Am*-FUC. Nevertheless, there was no significant difference between *Am*-FUC-treated and HFD-fed mice (Figure 2E). In the OGTT and IITT experiments (Figure 2F–I), *Am*-FUC markedly decreased the AUCs by 12.89 and 19.93% (*p* < 0.05), respectively. When administrated with *Am*-LFUCs, AUC_OGTT_ and AUC_IITT_ were significantly and sequentially decreased in the *Am*-LFUC1 group (19.13 and 24.50%, respectively, *p* < 0.05), *Am*-LFUC2 group (23.57 and 25.80%, respectively, *p* < 0.05), *Am*-LFUC3 group (29.24 and 31.88%, respectively, *p* < 0.05), and *Am*-LFUC4 group (38.67 and 39.40%, respectively, *p* < 0.05). Interestingly, AUC_OGTT_ in the *Am*-LFUC4 group was markedly lower than that of the *Am*-FUC group compared with HFD-fed mice (*p* < 0.05).

Insulin resistance preferentially occurred in the liver, and glycogen content and the related enzymes activities could give a visually direction. In the liver of insulin-resistant mice, glycogen content, and PK and HK activity were significantly enhanced by 1.29-fold, 41.57%, and 64.89% (*p* < 0.05), respectively, in the *Am*-FUC group, whereas GP and G6Pase activities were obviously lowered 20.47 and 24.55% (*p* < 0.05), respectively (Figure 2J–N). When treated with *Am*-LFUCs, the mice in the four *Am*-LFUC groups showed significant increases in hepatic glycogen contents by 1.59-, 1.86-, 2.43-, and 2.80-fold (*p* < 0.05), PK activities by 68.34, 84.32, 100.00%, and 1.14-fold (*p* < 0.05), HK activities by 84.04%, 1.11-, 1.37-, and 1.78-fold (*p* < 0.05). Moreover, GP and G6Pase activities were dramatically decreased (GP, 24.24, 24.96, 33.87, and 39.73%; G6Pase, 29.52, 35.24, 40.53, and 46.88%, respectively) in the four *Am*-LFUC groups compared with the HFD group. The effects of *Am*-LFUC4 on GP, PK, and G6Pase activities were superior to those of *Am*-FUC (*p* < 0.05). *Am*-LFUC4 resulted in higher HK activities than both *Am*-FUC and *Am*-LFUC1 (*p* < 0.05).

We also determined p-GS protein in the liver tissue and m-Glut4 protein in the skeletal muscle tissue using Western blotting. As shown in Figure 2O,P, *Am*-FUC and the four *Am*-LFUCs significantly increased p-GS protein expressions by 59.57%, 1.09-, 1.64-, 2.36-, and 2.96-fold (*p* < 0.05), respectively. Additionally, m-Glut4 protein expressions were also significantly increased by 0.94-, 2.11-, 3.89-, 4.39-, and 5.33-fold (*p* < 0.05) in *Am*-FUC- and its four depolymerized polysaccharide-treated mice. Interestingly, the effect of *Am*-LFUC4 on the p-GS protein was better than that of *Am*-FUC, *Am*-LFUC1, and *Am*-LFUC2 (*p* < 0.05), and *Am*-LFUC4 also exhibited higher efficacy on m-Glut4 protein than *Am*-FUC (*p* < 0.05).

The aforementioned results showed that *Am*-FUC and the four *Am*-LFUCs could improve insulin resistance, and the effects of *Am*-LFUCs were better than those of *Am*-FUC. Interestingly, *Am*-LFUC, with its smaller M_w_, exhibited stronger insulin resistance-alleviation activity, demonstrating that low M_w_ fucoidans are more effectively at mitigating insulin resistance than large M_w_ polysaccharides.

### 2.2. Effects of Fucoidans with Different C_s_ on Insulin Resistance

Sulfation is closely related to the bioactivity of fucoidans. The M_w_ and C_s_ of *Am*-FUC were 1614.1 ± 31.6 kDa and 26.3 ± 2.7%, while those of *Ht*-FUC were 1567.6 ± 34.1 kDa and 31.2 ± 1.6%, respectively, suggesting *Am*-FUC and *Ht*-FUC had similar M_w_, whereas the C_s_ of *Ht*-FUC was significantly higher due to one more sulfation in saccharide unit A (Figure 1). Therefore, we compared the insulin resistance-alleviating activities of *Am*-FUC and *Ht*-FUC, to determine the effect of C_s_ in fucoidans on insulin resistance.

**Figure 3:**
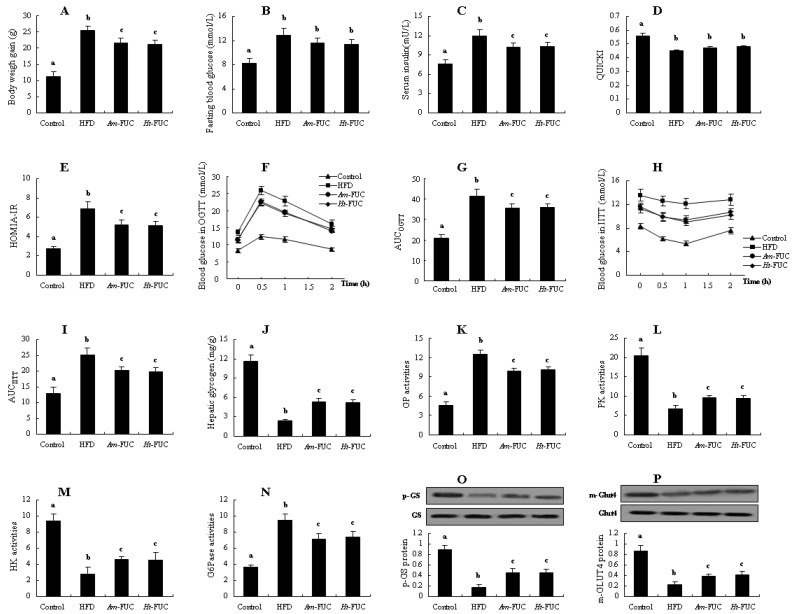
Comparatively analyzing the effects of between *Am*-FUC and *Ht*-FUC (similar molecular weight but different sulfation concentration) on alleviating insulin resistance in HFD-fed mice. *Acaudina molpadioides* fucoidans, *Am*-FUC; *Holothuria tubulosa* fucoidans, *Ht*-LFUC. (**A**), Body weight gain; (**B**), Fasting blood glucose; (**C**), Serum insulin; (**D**), HOMIA-IR value; (**E**), QUICKI score; (**F**), OGTT test; (**G**), AUC in OGTT test; (**H**), IITT test; (**I**), AUC in IITT test; (**J**), Hepatic glycogen; (**K**), GP activities; (**L**), PK activities; (**M**), HK activities; (**N**), G6Pase activities; (**O**), p-GS protein expression (p-GS and GS, 85 kDa); (**P**), m-Glut4 protein expression (m-Glut4 and Glut4, 43 kDa). Body weight gain was assessed using 12 mice. OGTT and IITT were assessed using 6 mice, respectively. p-GS protein was assessed using the 3 mice with 40 U/kg insulin stimulation while m-Glut4 protein as 0.5 U/kg insulin. The other parameters were assessed using the 6 mice treated with normal saline. Different lowercase letters represent significant difference (*p* < 0.05) compared between groups.

As shown in Figure 3, body weight gain was significantly decreased in the *Am*-FUC and *Ht*-FUC group by 14.74 and 17.03% (*p* < 0.05), respectively, compared with HFD-fed mice. *Am*-FUC and *Ht*-FUC both caused considerable reductions in serum insulin and HOMIA-IR score compared with the HFD group (serum insulin, 14.57 vs. 14.32%; HOMIA-IR, 23.80 vs. 24.53%, respectively, *p* < 0.05), while neither of them significantly affected fasting blood glucose and QUICKI value. OGTT and IITT data indicated that *Am*-FUC and *Ht*-FUC had the quite blood glucose levels and similar AUC (OGTT, 14.46 vs. 13.76%; IITT, 19.93 vs. 20.81%, respectively, *p* < 0.05). Moreover, hepatic glycogen contents, and PK and HK activities were all significantly elevated in the two fucoidan-treated mice compared with the insulin-resistant animals (hepatic glycogen, 1.29 fold vs. 1.22 fold; PK, 41.57 vs. 39.31%; HK, 64.89 vs. 62.99%, respectively), while GP and G6Pase activities were both remarkably lowered (GP, 20.47 vs. 19.18%; G6Pase, 24.55% vs. 23.12%, respectively). Furthermore, the mice administrated with *Am*-FUC and *Ht*-FUC showed significant increases in p-GS protein expression in the liver by 1.71 fold and 1.65 fold, and m-Glut4 protein expression in the skeletal muscle by 69.57 and 73.91%, respectively. These results implied that *Am*-FUC and *Ht*-FUC exerted similar insulin resistance-alleviating activity, indicating that C_s_ had little impact on fucoidan-improved insulin resistance. However, these results were not persuasive because sulfation is indeed a pivotal functional group in fucoidans. Thus, further investigation is needed.

**Figure 4:**
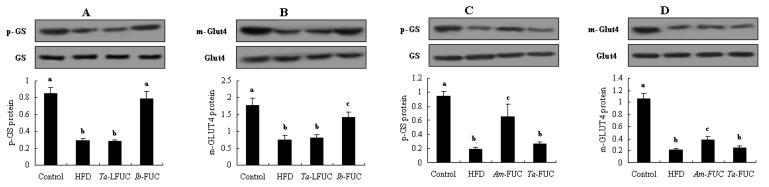
Comparatively analyzing the effects of between *Ta*-LFUC and *Ib*-FUC (a similar molecular weight but different sulfation concentration), *Ta*-LFUC with sulfation at ortho-fucose and *Am*-FUC with sulfation at meta-position (quite molecular weight and sulfation concentration) on hepatic p-GS protein expression and skeletal muscle m-Glut4 protein expression in HFD-fed mice. *Acaudina molpadioides* fucoidans, *Am*-FUC; *Isostichopus badionotus* fucoidans, *Ib*-LFUC; *Thelenota ananas* fucoidans, *Ta*-LFUC. (**A**), p-GS protein expression (*Ta*-LFUC vs. *Ib*-FUC; p-GS and GS, 85 kDa); (**B**), m-Glut4 protein expression (*Ta*-LFUC vs. *Ib*-FUC; m-Glut4 and Glut4, 43 kDa); (**C**), p-GS protein expression (*Ta*-FUC vs. *Am*-FUC); (**D**), m-Glut4 protein expression (*Ta*-FUC vs. *Am*-FUC). p-GS protein was assessed using the 3 mice with 40 U/kg insulin stimulation while m-Glut4 protein as 0.5 U/kg insulin. Different lowercase letters represent significant difference (*p* < 0.05) compared between groups.

In the following experiments, we comparatively investigated the effects of *Ta*-LFUC and *Ib*-FUC in alleviating insulin resistance. The two fucoidans had similar M_w_ (450.7 ± 15.1 vs. 435.3 ± 13.4 kDa) and distinguished C_s_ (28.4 ± 3.9 vs. 32.9 ± 3.2%). Results are presented in Table 1 and Figure 4. Both fucoidans significantly reduced body weight gain by 9.40 and 37.32% (*p* < 0.05, Table 1), respectively. However, only *Ib*-FUC remarkably decreased body weight gain, fasting blood glucose, serum insulin, HOMIA-IR score, and increased QUICKI value relative to HFD mice (*p* < 0.05). There was no significant difference in these five insulin resistance-related parameters between the *Ta*-LFUC and HFD groups (*p* > 0.05). In OGTT, *Ta*-LFUC-treated mice showed marked reductions in blood glucose at 1 and 2 h compared with HFD-fed mice, while the mice in the *Ib*-FUC group exhibited noticeable decreases throughout the OGTT process. The reductions in AUC_OGTT_ attributed to *Ta*-LFUC and *Ib*-FUC relative to the HFD group were 10.69 and 36.66%, respectively. In IITT, *Ib*-FUC resulted in conspicuous improvements in blood glucose and AUC_IITT_ (*p* < 0.05), while *Ta*-LFUC had no significant effect. Moreover, as shown in Table 1, in the liver tissues, glycogen content and glycometabolism-related enzymatic activities were no significantly altered. In contrast, the mice in the *Ib*-FUC group experienced dramatic increases in hepatic glycogen content, and PK and HK activities by 2.75-, 1.25-, and 1.67-fold (*p* < 0.05) compared with HFD-fed mice, as well as considerable decreases in GP and G6Pase activities by 35.15 and 45.61% (*p* < 0.05), respectively. Additionally, Figure 4A,B show that *Ib*-FUC could dramatically enhance p-GS protein expression in the liver and m-Glut4 protein expression in the skeletal muscle in insulin-resistant mice (*p* < 0.05). In contrast, p-GS and m-Glut4 protein expression were not significantly altered in the *Ta*-LFUC group compared with the HFD group. Evidence shows that the effects of *Ib*-FUC with higher C_s_ were superior to those of *Ta*-LFUC with lower C_s_, suggesting that sulfation is vital for fucoidans in mitigating insulin resistance.

### 2.3. Effects of Sulfation at Ortho-, or Meta-fucose Positions on Insulin Resistance

In the prior experiments, without considering the effects of M_w_, *Ht*-FUC bioactivity, with higher C_s_, was equated to *Am*-FUC bioactivity, with lower C_s_. However, the activity of *Ib*-FUC with higher C_s_ was superior to that of *Ta*-LFUC with lower C_s_. These seemingly exhibited contradictory results. As shown in Figure 1, the structure of *Ht*-FUC had 2,4-dual-sulfation (2,4DS) in fucose A, 2-sulfation (2S) in fucose C, and 2S in fucose D, whereas *Am*-FUC had 2,4DS in fucose A and 2S in fucose C, showing that there was one more 2S in the fucose neighboring 2,4DS-fucose in *Ht*-FUC than in *Am*-FUC. The structure of *Ib*-FUC had 2,4DS in fucose A, 2S in fucose B, and 2S in fucose C, whereas *Ta*-FUC had 2,4DS in fucose A and 2S in fucose B. There was one more 2S in fucose spaced 2,4DS-fucose in *Ib*-FUC than in *Ta*-FUC. In other words, the fucoses with sulfation in *Am*-FUC were meta-positioned, while in *Ta*-FUC, they were ortho-positioned. Thus, we speculated that the contradictory results might be related to sulfation at ortho- or meta-fucose. The subsequent experiments were conducted to verify our speculation through comparative analysis of the effects of *Am*-FUC and *Ta*-FUC (M_w_, 1614.1 ± 31.6 vs. 1380.0 ± 8.1 kDa; C_s_, 26.3 ± 2.7 vs. 28.2 ± 3.5%, respectively) on insulin resistance alleviation.

As shown in Table 1, there were no significant differences in body weight gain, fasting blood glucose, serum insulin, HOMIA-IR, and QUICKI values between HFD and *Ta*-FUC groups, while body weight gain, serum insulin, and HOMIA-IR score were remarkably improved in the insulin-resistant mice when treated with *Am*-FUC (*p* < 0.05). With regards to OGTT and IITT, the blood glucose levels at all time points did not significantly differ between the *Ta*-FUC and HFD groups: similar findings were observed for AUC_OGTT_ and AUC_IITT_. In contrast, *Am*-FUC caused apparent decreases in blood glucose levels at all time points in OGTT and IITT compared with HFD-fed mice (*p* < 0.05), as well as distinct reductions in AUC_OGTT_ and AUC_IITT_ (*p* < 0.05). Moreover, hepatic glycogen content and glucose metabolism-related enzyme activities were also unchanged in insulin-resistant mice when administrated with *Ta*-FUC. Comparatively, *Am*-FUC noticeably enhanced glycogen content and PK and HK activities, and inhibited GP and G6Pase activities in the liver of HFD-fed mice (*p* < 0.05). Furthermore, as shown in Figure 4C,D, *Am*-FUC caused significant increases in p-GS protein expression in the liver and m-Glut4 protein in the skeletal muscle of insulin-resistant mice (*p* < 0.05), while *Ta*-FUC had no effect. These results visibly showed that *Am*-FUC could improve insulin resistance while *Ta*-FUC could not, indicating that sulfation at meta-fucose is essential for fucoidans to alleviate insulin resistance.

### 2.4. Effects of 2-O-Sulfation or 4-O-Sulfation on Insulin Resistance

**Figure 5:**
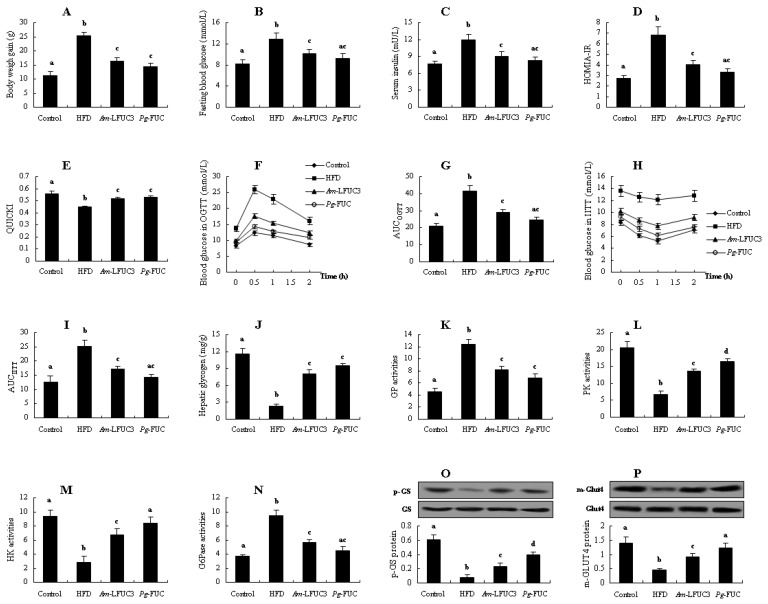
Comparatively analyzing the effects of between *Am*-LFUC3 with 2-*O*-sulfation and *Pg*-FUC with 4-*O*-sulfation (quite molecular weight and sulfation concentration) on alleviating insulin resistance in HFD-fed mice. *Acaudina molpadioides* fucoidans, *Am*-FUC; *Pearsonothuria graeffei* fucoidans, *Pg*-LFUC. (**A**), Body weight gain; (**B**), Fasting blood glucose; (**C**), Serum insulin; (**D**), HOMIA-IR value; (**E**), QUICKI score; (**F**), OGTT test; (**G**), AUC in OGTT test; (**H**), IITT test; (**I**), AUC in IITT test; (**J**), Hepatic glycogen; (**K**), GP activities; (**L**), PK activities; (**M**), HK activities; (**N**), G6Pase activities; (**O**), p-GS protein expression (p-GS and GS, 85 kDa); (**P**), m-Glut4 protein expression (m-Glut4 and Glut4, 43 kDa). Body weight gain was assessed using 12 mice. OGTT and IITT were assessed using 6 mice, respectively. p-GS protein was assessed using the 3 mice with 40 U/kg insulin stimulation while m-Glut4 protein as 0.5 U/kg insulin. The other parameters were assessed using the 6 mice treated with normal saline. Different lowercase letters represent significant difference (*p* < 0.05) compared between groups.

Sulfation site may affect fucoidan activity. Thus, we investigated the effects of *Am*-LFUC3 (with a 2-*O*-sulfation; M_w_, 327.2 ± 12.5 kDa; C_s_, 26.8 ± 2.6%) and *Pg*-FUC (with a 4-*O*-sulfation; M_w_, 310.5 ± 26.8 kDa; C_s_, 27.4 ± 2.5%) on insulin resistance. As shown in Figure 5, both *Am*-LFUC3 and *Pg*-FUC significantly lowered body weight gain (by 36.02 vs. 43.33%), fasting blood glucose (by 21.02 vs. 28.01%), serum insulin (by 24.81 vs. 31.39%), HOMIA-IR value (by 41.12 vs. 51.24%), and enhanced QUICKI score (by 15.56 vs. 17.78%, respectively) compared with HFD-fed mice. Similarly, *Am*-LFUC3 and *Pg*-FUC obviously enhanced glucose tolerance and insulin tolerance, showing AUC_OGTT_ 30.52 vs. 41.22%, and AUC_IITT_ 31.88 vs. 43.52%. Alhough there were no significant differences in the aforementioned parameters between the two groups, the *Pg*-FUC group data were more tended towards that of the control group more than that of the *Am*-LFUC3 group. In the liver tissue, *Am*-LFUC3 and *Pg*-FUC remarkably elevated hepatic glycogen contents (2.43- vs. 3.09-fold), PK activities (1.00- vs. 1.45-fold), and HK activities (1.37- vs. 1.98-fold), while the two fucoidans decreased GP activities (33.87 vs. 45.26%) and G6Pase (40.53 vs. 52.28%, respectively). Western blotting results showed that *Am*-LFUC3 and *Pg*-FUC caused p-GS protein expression increases by 2.01- and 3.88-fold, respectively, in the liver of insulin-resistant mice, as well as m-Glut4 protein expression increases by 1.00- and 1.67-fold, respectively, in skeletal muscle. Interestingly, PK and HK activities, p-GS and m-Glut4 protein expressions in the *Pg*-FUC group were remarkably elevated compared with these parameters in the *Am*-LFUC3 group (*p* < 0.05). The aforementioned results showed that the bioactivity of *Pg*-FUC were superior to that of *Am*-LFUC3, suggesting that fucoidans with 4-*O*-sulfation make a higher contribution to insulin resistance alleviation than fucoidans with 2-*O*-sulfation.

## 3. Discussion

Our previous studies also indicated that fucoidans derived from *Acaudina molpadioides* and *Isostichopus badionotus* could improve insulin resistance [18,19,24]. However, Wright et al. found that there were no significant changes in insulin resistance parameters in obese humans aged between 18 and 65 years when treated with 500 mg fucoidans per day for 90 days [25]. Taken together, these studies imply that different fucoidans may exert different effects on insulin resistance. However, it was unclear which fucoidan structures or functional groups influenced the observed effects. In the present study, we investigated the role of the structure–activity relationship of SC-FUC in improving in insulin resistance. Our data indicated that low M_w_, high C_s_, sulfation at meta-fucose, and 4-*O*-sulfation were beneficial for improving insulin resistance.

M_w_ of fucoidans has been reported to be related to their biological activities. Fucoidans with lower M_w_ have certain advantages over those with higher M_w_ due to their improved diffusion into biological tissues and the bloodstreams [12]. Zhu et al. reported that the anti-obesity activity of depolymerized sulfated polysaccharides (14.3 kDa) from the sea cucumber, *Stichopus japonicus*, was more effective than that of polysaccharides (fucoidans >670 kDa) in HFD-fed mice [26]. These depolymerized sulfated polysaccharides were more amenable to decreasing fat accumulation than sulfated polysaccharides, but body weight gain and insulin resistance parameters (such as AUC_OGTT_ and HOMIA-IR) did not change significantly between the two groups. Yang et al. reported that the anticancer activity of fucoidans could be significantly enhanced by lowering their M_w_ [27]. In the present study, although there were no significant changes in fasting blood glucose, serum insulin, and AUC_IITT_ between *Am*-FUC and *Am*-LFUCs groups, a remarkable increase in QUICKI and reductions in body weight gain, AUC_OGTT_, and HOMIA-IR were apparent in *Am*-LFUC4-treated animals compared with *Am*-FUC-fed mice. Similar changes were exhibited in several insulin resistance parameters in the liver of HFD-fed mice. These demonstrated that M_w_ could affect SC-FUC activity, lower M_w_ of SC-FUC with stronger effects on improving insulin resistance. Fucoidan activity became increasingly apparent as M_w_ decreased.

Nonetheless, some reports contradict our findings. Xu et al. reported that *Ta*-FUC and their depolymerized derivatives could significantly prevent ethanol-induced gastric ulcers, including anti-oxidations and anti-inflammation, but there were no significant changes between *Ta*-FUC (1380.0 kDa) and *Ta*-LFUCs (828.7, 483.0, and 215.0 kDa) [16]. In the present study, we also investigated the effects of *Ta*-FUC on alleviating insulin resistance, and the results showed no biological activity of the fucoidans in HFD-fed mice. Fucoidans derived from brown algae with different M_w_ were also had their bioactivities investigation. In contrast to our results, the relationship between the M_w_ of fucoidans and their antioxidant or epithelial–mesenchymal transition activities is not simply linear. These discrepancies may result from fucoidans isolated from the different species and different M_w_ distributions (this study M_w_ 140–1600 kDa, fucoidans from brown alga M_w_ 1–60 kDa) [28,29].

Sulfation is a critical determinant of fucoidan bioactivity, involving both the C_S_ and sulfate position. Considering similar M_w_ but varying C_S_ in *Am*-FUC and *Ht*-FUC, we comparatively analyzed their effects to investigate the influence of C_S_ influence. The C_S_ of *Ht*-FUC was higher than that of *Am*-FUC (*Ht*-FUC having one more sulfate at fucose A), but the effects of *Ht*-FUC on improving insulin resistance were not superior to those of *Am*-FUC. This seemingly implied that C_S_ did not play a crucial role in the ability of fucoidans to alleviate insulin resistance. However, several studies opposed to these findings. For example, Li et al. reported that fucoidans from *Sargassum fusiforme* with 47.5 kDa M_w_ and 20.8% C_S_ exerted an inhibitory effect on HMEC-1 cell angiogenesis. However, the lower C_S_ fractions (7.5% C_S_ and 12.4 kDa M_w_) had no effect [30]. The over-sulfation of fucoidans through chemical modification resulted in a stronger inhibition of cancer cell growth and angiogenesis [31,32]. All these papers vehemently suggested the importance of fucoidan C_S_ on their bioactivity, which conflicted with our aforementioned results. To clarify whether C_S_ of SC-FUC could affect insulin-resistant activity, further investigations were conducted using *Ta*-LFUC and *Ib*-FUC. The two fucoidans have similar M_w_, but *Ib*-FUC had a higher C_S_. *Ta*-LFUC failed to improve HFD-induced insulin resistance. Comparatively, *Ib*-FUC significantly mitigated insulin resistance. These results implied that higher C_S_ might be beneficial for fucoidans in improving insulin resistance.

Exclusive M_w_ influence, sulfation position would be the only explanation for our observed findings. C_S_ was higher in *Ht*-FUC than in *Am*-FUC, because there was one more sulfate substitution in the fucose neighboring 2,4DS-fucose in *Ht*-FUC, unlike in *Am*-FUC. Considering the similar bioactivities of *Ht*-FUC and *Am*-FUC, it was speculated that the 2S in the fucose neighboring 2,4DS-fucose was dispensable for fucoidan bioactivity. On the other hand, *Ib*-FUC possessed one more sulfation, located in the fucose spaced 2,4DS-fucose compared with *Ta*-FUC. The effects of *Ib*-FUC were superior to those of *Ta*-LFUC. Thus, 2S in fucose spaced 2,4DS-fucose might be indispensable for fucoidans to improve insulin resistance. To confirm the hypothesis, the bioactivities of *Am*-FUC (with meta-fucose sulfate substitution) and *Ta*-FUC (with ortho-fucose sulfate substitution) were compared. All the insulin resistance-related parameters indicated that *Am*-FUC bioactivity exceeded that of *Ta*-FUC. These observations demonstrated that sulfation at meta-fucose was vital for SC-FUC insulin resistance-alleviating effect. However, it is regrettable that there are currently no relevant studies to support our conclusion.

2-*O*-sulfation and 4-*O*-sulfation were identified in SC-FUC. Recently, researches have reported that the 4-*O*-sulfation substitution pattern in SC-FUC exhibited more potent effects on anti-inflammatory and anti-hyperlipidemia activities [22,33]. The authors comparatively investigated the bioactivities of *Ib*-FUC and *Pg*-FUC, and found that the effects of *Pg*-FUC with 4-*O*-sulfation were stronger than those of *Ib*-FUC with 2-*O*-sulfation. However, the C_S_ in the two SC-FUC were not similar because there was one more 2-*O*-sulfation in the fucose neighboring 2,4DS-fucose in *Ib*-FUC than in *Pg*-FUC. Therefore, the consequences could be controversial.

In the present study, the effects of *Ib*-FUC and *Am*-LFUC3 on alleviating insulin resistance were contrastively determined. The two fucoidans have the same M_w_ and C_S_, while the difference was 4-*O*-sulfation in *Pg*-FUC and 2-*O*-sulfation in *Am*-LFUC3. PK and HK activities and p-GS and m-Glut4 protein expressions were considerably improved in the *Pg*-FUC group than in the *Am*-LFUC3 group. Moreover, there were no significant differences in the other insulin resistance-related parameters between the two SC-FUC groups, but the data of the *Pg*-FUC group were closer to those of the control group. The greater effects of *Pg*-FUC (vs. *Am*-LFUC3) indicated that 4-*O*-sulfation in SC-FUC was more favorable for insulin resistance mitigation than 2-*O*-sulfation. Several researches supported our results. For instance, Li et al. stated that the 4-*O*-sulfated structure of *Pg*-FUC might be the primary functional group to treat with metabolic syndromes [34]. Additionally, Mandal et al. founded that sulfate groups located at C-4 of (1→3)-linked fucopyranosyl units (4-*O*-sulfation) appeared to be especially important for the anti-herpetic activity of brown seaweed fucoidans [35]. Fereira et al. indicated that both 2,4-di-*O*-sulfation and signal 4-*O*-sulfation of fucoidans had a distinguished potent anticoagulant activity, while exclusively 2-*O*-sulfation of fucoidans was almost devoid of activity [36]. Fonseca et al. also proved the importance of 2,4-di-*O*-sulfation in fucoidans with anticoagulant activity [37]. On the contrary, Chevolot et al. reported that anticoagulant activity was greatly magnified upon treatment with fucoidans containing 2-*O*-sulfation and 3-*O*-sulfation [38].

## 4. Materials and Methods 

### 4.1. Preparation of Fucoidans

Dried sea cucumbers, *Acaudina molpadioides* (*Am*), *Isostichopus badionotus* (*Ib*), *Thelenota ananas* (*Ta*), *Holothuria tubulosa* (*Ht*), and *Pearsonothuria graeffei* (*Pg*) were procured from marine product markets in Zhoushan, China. The species were identified by Professor Laijin Su of Wenzhou University (Wenzhou, China). Fucoidans were prepared as previously reported [13]. Their structures, M_w_, and C_s_ are shown in Figure 1 [33,39].

### 4.2. Low-M_w_ Fucoidans Preparation

Different low-M_w_ fucoidans from *Acaudina molpadioides* (*Am*-LFUC) were obtained according to the method described by Yu et al [23]. Briefly, the intracellular enzyme Flavobacteriaceae CZ1127 (from marine bacterial stain *W. fucanilytica*) was added to an *Am*-FUC solution (20 mmol/L Tris-HCl and 0.3 mol/L NaCl) for enzymatic hydrolysis at 37 °C for 5, 7, 9, and 11 h. After thermal inactivation at 100 °C, the enzymatic solution was filtered through a Sephacryl S-300/400/500 column to collect the purified *Am*-LFUC. Their average M_w_ were determined by high performance size exclusion chromatography-multiangle laser light scattering (HPSEC-MALLS), which consisted of HPLC, refractive index detection (Agilent 1260, Aglient, Santa Clara, CA, USA), 18-angle laser light scattering instrument (Dawn HELEOS, Wyatt, SB, USA), and SEC column. The mobile phase was 0.1 M Na_2_SO_4_. The C_s_ were determined using a Dionex IC-2000 ion chromatography (Dionex, Sunnyvale, CA, USA). Briefly, after hydrolysis with trifluoroacetic acid and drying under vacuum, 20 µL fucoidan hydrolysate was injected into the ion chromatograph to determine the conductivity signal. The chromatographic system included a Shodex IC SI-90E separation column (250 mm × 4.6 mm; Showa Denko America, Inc., New York, NY, USA), and the eluent was 1 mM Na_2_CO_3_ + 4 mM NaHCO_3_ with 2.5% acetone. A standard sulfate was used to quantify the sulfate content of fucoidans according to the peak area. In these experiments, we collected four different *Am*-LFUC with M_w_ and C_s_ as follows: *Am*-LFUC1 (M_w_ 847.5 ± 22.4 kDa, C_s_ 26.6 ± 1.8%), *Am*-LFUC2 (M_w_ 530.9 ± 13.0 kDa, C_s_ 27.1 ± 2.5%), *Am*-LFUC3 (M_w_ 327.2 ± 12.5 kDa, C_s_ 26.8 ± 2.6%), and *Am*-LFUC4 (M_w_ 142.4 ± 13.3 kDa, C_s_ 27.2 ± 2.8%). *Am*-FUC and the depolymerized *Am*-LFUC had the similar sulfate contents, indicating that sulfate groups were not hydrolyzed during degradation.

Using the foregoing methods, *Ta*-FUC was subjected to enzymolysis using Flavobacteriaceae CZ1127 for 10 h, and a low M_w_ *Ta*-FUC (*Ta*-LFUC) was finally obtained with M_w_ and C_s_, of 450.7 ± 15.1 kDa and 28.4 ± 3.9%, respectively. Yu et al. have proved that structural features of *Ta*-FUC were retained during enzymatic degradation [23].

### 4.3. Animal Experiments

Male C57BL/6J mice (licensed ID: SCXK2019-0001), 16–18 g, were purchased from Vital River Laboratory Animal Center (Beijing, China). The mice were kept in a stable environment under a 12-h light/dark cycle daily throughout the experiment. The mice (144 in total and 12 animals in each group) were assigned to 12 groups: control group (normal chow diet feeding: 70% carbohydrate, 20% protein, and 10% fat), HFD group (HFD feeding: 29% carbohydrates, 16% protein, and 55% fat), and 10 experiment groups (administered with HFD as well as 40 mg/kg of 5 types of fucoidans, 4 types of *Am*-LFUC, or *Ta*-LFUC intragastrically, 0.2 mL daily). Animal experiments were conducted for 12 w. At 11 w of feeding, an oral glucose tolerance test (OGTT, six mice per group) and an intraperitoneal insulin tolerance test (IITT, six mice per group) were conducted. At 12 w of administration, all mice were sacrificed. Blood was collected to test insulin resistance-related parameters. The liver and skeletal muscle tissues were collected for further study. 

Firstly, *Am*-FUC and the 4 depolymerized *Am*-LFUCs groups were used to analyze the activities of different M_w_ fucoidans. Secondly, we investigated the influence of different C_s_ fucoidans using comparative analysis in between *Am*-FUC and *Ht*-FUC groups, and *Ta*-LFUC and *Ib*-FUC groups (they have different C_s_ while similar M_w_). Subsequently, to clarify the effects of sulfation at ortho- or meta-fucose, a comparative analysis was studied in between *Am*-FUC and *Ta*-FUC groups (they have similar M_w_ and C_s_, but sulfation at ortho-fucose in *Ta*-FUC and sulfation at meta-fucose in *Am*-FUC). Finally, we investigated the different effects of 2-*O*-sulfation and 4-*O*-sulfation using *Am*-LFUC3 and *Pg*-FUC groups (they have similar M_w_, C_s_, and sulfation at meta-fucose, while a 2-*O*-sulfation in *Am*-LFUC3 and 4-*O*-sulfation in *Pg*-FUC).

### 4.4. OGTT and IITT

At 11 w of treatment, 5-h fasted mice were intragastrically administered with 2 g/kg glucose, and blood glucose was measured using a commercial kit (Biosino, Beijing, China) at 0, 0.5, 1, and 2 h for OGTT (six mice per group). Similarly, another six mice were intraperitoneally injected with 0.5 U/kg insulin, and blood glucose was measured at the aforementioned time points. Equation (1) was used to calculate the areas under the curve of OGTT (AUC_OGTT_) and IITT (AUC_IITT_) [40].

AUC_OGTT_/AUC_IITT_ = 0.25 × A + 0.5 × B + 0.75 × C + 0.5 × D (1)

where A, B, C, and D represent blood glucose level at 0, 0.5, 1, and 2 h after treating with glucose or insulin, respectively.

### 4.5. Insulin Stimulation and Plasma Membrane Preparation

At the end of the animal experiments, the mice were sacrificed after treatment with normal saline (six mice in each group) or insulin stimulation for p-GS protein (three animals per group) and plasma membrane Glut4 4 (m-Glut4) protein (three animals each). Briefly, 5 min after intraperitoneal injection with 40 U/kg insulin, the mice were sacrificed to collect the liver tissue to measure p-GS protein. Meanwhile, another three mice were sacrificed 30 min after 0.5 U/kg insulin injection, and the skeletal muscle was collected to measure m-Glut4 protein.

Plasma membrane from skeletal muscle was extracted according to our previous study [41]. Briefly, skeletal muscle from the 0.5 U/kg insulin-injected mice was homogenized to extract the crude membrane through triple centrifugation at 1200, 9000, and 19,000× *g*. A sucrose density-gradient (25, 32, and 35%) centrifugation at 150,000× *g* for 16 h was carried out to separate the plasma membrane. The 25% sucrose fraction was collected and centrifuged at 190,000× *g* for 1 h to obtain the Glut4 protein at m-Glut4.

### 4.6. Blood Glucose and Plasma Insulin Analysis

At the end of animal experiments, the mice were sacrificed to collect blood. Fasting blood glucose levels were determined using glucose testing kits (Biosino, Beijing, China), and serum insulin levels were determined using insulin ELISA kits (Invitrogen, Carlsbad, CA, USA). Equations (2) and (3) were used to calculate the homeostasis model assessment of insulin resistance index (HOMA-IR) and quantitative insulin sensitivity check index (QUICKI), respectively [42].

HOMA-IR = (fasting blood glucose × serum insulin)/22.5 (2)

QUICKI = 1/[log(fasting blood glucose) + log(serum insulin)] (3)

### 4.7. Hepatic Glycogen Content Detection

The liver tissues (0.1 g) were homogenized using 4 M NaOH and heated in boiling water. After diluting with distilled water, the homogenate was centrifuged to collect the pellet. Hepatic glycogen content was determined form the pellet dissolved in 1.5 mL distilled water using a commercial kit (Jiancheng, Nanjing, Jiangsu, China).

### 4.8. Glucose Metabolism Enzymes Test

The activities of glucose metabolism-related enzymes, including GP, PK, HK, and G6Pase in the liver of normal saline-treated mice, were assayed according to our previous study’s method [16]. Briefly, HK, PK, and GP activities were determined using liver homogenate in Tris-HCl (Ph 7.4), while G6Pase activity was determined in HCl-imidazole (pH 6.5). Total protein content was determined using a commercial kit (Biosino). HK activity was determined using glucose 6-phosphate dehydrogenase, PK activity using L-phosphate dehydrogenase, GP using phosphoglucomutase and glucose-6-phosphatase, G6Pase using mutarotase and dehydrogenase. After reacting at 37 °C for 2 min, the system was tested for OD values at 340 nm. The enzyme activities were calculated according to Equation (4) [24].

Enzyme activities = OD_340nm_ every minute/(6.22 × total protein content) (4)

### 4.9. Western Blotting

The liver and skeletal muscle tissues stimulated using 40 and 0.5 U/kg insulin, respectively, were homogenized in IP lysis buffer. The proteins, including m-Glut4 protein, were separated using 10% SDS-PAGE and subsequently electro-transferred onto PVDF membranes. The membranes were incubated with diluted Glut4 (1:500), p-GS (1:200), or GS (1:200) antibodies (Cell Signaling, Danvers, MA, USA), followed by incubation with HRP conjugated IgG antibody. The bands were visualized using an ECL kit. Normalization of p-GS and m-Glut4 protein expressions were controlled by total GS and Glut4 protein.

### 4.10. Statistical Analysis

Data were evaluated using a one-way analysis of variance followed by Duncan’s test using SPSS version 17.0 (SPSS Inc., Chicago, IL, USA). Values of *p* < 0.05 were considered statistically significant.

## 5. Conclusions

**Figure 6:**
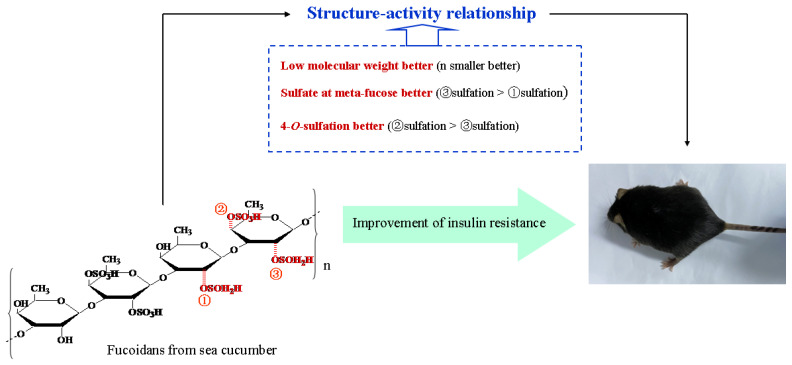
The structure–activity relationship of fucoidans from sea cucumber on improvement of insulin resistance. Our data showed that low molecular, sulfate at meta-fucose, and 4-*O*-sulfation played a significant part in the insulin resistant alleviation of fucoidans in mice.

The present study clarified the structure–activity relationship of SC-FUC in ameliorating insulin resistance in HFD-fed mice (Figure 6). SC-FUC with lower M_w_ can more effectively mitigate insulin resistance than polysaccharides with larger M_w_. Higher C_S_ contribute more to SC-FUC bioactivity. Sulfation position is vital for SC-FUC in mitigating insulin resistance, showing that meta-fucose of sulfate substitution is superior to ortho-fucose, and 4-*O*-sulfation is more potent than 2-*O*-sulfation.

## Figures and Tables

**Table 1:** Comparison analysis on several insulin resistance-related parameters between *Ta*-LFUC and *Ib*-FUC, *Ta*-FUC and *Am*-FUC in HFD mice.

		Control	HFD	Ta-LFUC	Ib-FUC	Ta-FUC	Am-FUC
Body weight gain (g)		11.16 ± 1.47 ^a^	25.43 ± 1.25 ^b^	23.04 ± 1.33 ^b^	15.94 ± 1.41 ^d^	24.37 ± 1.72 ^b^	21.68 ± 1.39 ^c^
Fasting blood glucose (mmol/L)		8.25 ± 0.77 ^a^	12.89 ± 1.13 ^b^	12.49 ± 0.66 ^b^	9.61 ± 0.59 ^d^	13.24 ± 0.71 ^b^	11.57 ± 0.84 ^b^
Serum insulin (mU/L)		7.63 ± 0.60 ^a^	12.01 ± 0.96 ^b^	11.12 ± 0.78 ^b^	8.75 ± 0.80 ^d^	11.87 ± 0.78 ^b^	10.26 ± 0.58 ^c^
HOMIA-IR		2.76 ± 0.21 ^a^	6.85 ± 0.74 ^b^	6.16 ± 0.63 ^b^	3.82 ± 0.31 ^d^	6.95 ± 0.58 ^b^	5.22 ± 0.50 ^c^
QUICKI		0.56 ± 0.02 ^a^	0.45 ± 0.00 ^b^	0.47 ± 0.02 ^b^	0.52 ± 0.01 ^d^	0.46 ± 0.01 ^b^	0.47 ± 0.01 ^b^
OGTT	Blood glucose at 0 h (mmol/L)	8.22 ± 0.64 ^a^	13.57 ± 0.90 ^b^	12.26 ± 0.64 ^b^	9.33 ± 0.68 ^d^	13.30 ± 0.77 ^b^	11.42± 0.71 ^c^
	Blood glucose at 0.5 h (mmol/L)	12.24 ± 0.79 ^a^	25.88 ± 1.25 ^b^	24.25 ± 0.79 ^b^	15.56 ± 0.77 ^d^	24.94 ± 0.96 ^b^	22.15 ± 0.87 ^c^
	Blood glucose at 1 h (mmol/L)	11.47 ± 0.77 ^a^	22.87 ± 1.43 ^b^	20.04 ± 0.51 ^c^	13.68 ± 0.72 ^d^	22.51 ± 0.76 ^b^	19.02 ± 0.64 ^c^
	Blood glucose at 2 h (mmol/L)	8.53 ± 0.60 ^a^	16.07 ± 1.19 ^b^	14.17 ± 0.71 ^c^	11.85 ± 0.64 ^d^	15.72 ± 0.84 ^b^	14.64 ± 0.81 ^c^
	AUC_OGTT_	21.04 ± 1.67 ^a^	41.52 ± 3.21 ^b^	37.08 ± 2.56 ^c^	26.30 ± 1.81 ^d^	40.54 ± 3.06 ^b^	35.16 ± 2.24 ^c^
IITT	Blood glucose at 0 h (mmol/L)	8.27 ± 0.45 ^a^	13.57 ± 0.96 ^b^	12.51 ± 0.80 ^b^	9.53 ± 0.58 ^d^	13.17 ± 0.86 ^b^	11.60 ± 0.56 ^c^
	Blood glucose at 0.5 h (mmol/L)	6.09 ± 0.38 ^a^	12.56 ± 0.78 ^b^	11.66 ± 0.73 ^b^	8.01 ± 0.73 ^d^	12.42 ± 0.85 ^b^	9.81 ± 0.63 ^c^
	Blood glucose at 1 h (mmol/L)	5.19 ± 0.42 ^a^	12.13 ± 0.87 ^b^	11.17 ± 0.70 ^b^	7.30 ± 0.69 ^d^	11.89 ± 0.89 ^b^	9.38 ± 0.66 ^c^
	Blood glucose at 2 h (mmol/L)	7.07 ± 0.53 ^a^	12.80 ± 0.95 ^b^	11.86 ± 0.81 ^b^	8.42 ± 0.61 ^d^	12.55 ± 0.78 ^b^	10.63 ± 0.57 ^c^
	AUC_IITT_	12.54 ± 1.83 ^a^	25.17 ± 2.26 ^b^	23.27 ± 1.49 ^b^	16.07 ± 0.26 ^d^	24.70 ± 1.70 ^b^	20.16 ± 1.24 ^c^
Hepatic glycogen (mg/g)		11.59 ± 0.98 ^a^	2.34 ± 0.30 ^b^	3.17 ± 0.25 ^b^	8.77 ± 0.42 ^d^	2.88 ± 0.57 ^b^	5.35 ± 0.47 ^c^
Hepatic GP activities		4.58 ± 0.51 ^a^	12.46 ± 0.73 ^b^	11.24 ± 0.39 ^b^	8.08 ± 0.34 ^d^	11.61 ± 0.53 ^b^	9.91 ± 0.45 ^c^
Hepatic PK activities		20.44 ± 1.97 ^a^	6.76 ± 0.89 ^b^	7.25 ± 0.83 ^b^	15.20 ± 0.93 ^d^	6.57 ± 0.88 ^b^	9.57 ± 0.60 ^c^
Hepatic HK activities		9.39 ± 0.86 ^a^	2.82 ± 0.84 ^b^	3.35 ± 0.93 ^b^	7.53 ± 0.88 ^d^	3.04 ± 0.75 ^b^	4.65 ± 0.31 ^c^
Hepatic G6Pase activities		3.67 ± 0.24 ^a^	9.45 ± 0.83 ^b^	8.62 ± 0.85 ^b^	5.14 ± 0.77 ^d^	9.38 ± 0.55 ^b^	7.13 ± 0.70 ^c^

Data are presented as mean ± SD. Multiple comparisons were done using one way ANOVA. *Acaudina molpadioides* fucoidans, *Am*-FUC; *Isostichopus badionotus* fucoidans, *Ib*-LFUC; *Thelenota ananas* fucoidans, *Ta*-LFUC. Body weight gain was assessed using 12 mice. OGTT and IITT were assessed using 6 mice, respectively. The other parameters were assessed using the 6 mice treated with normal saline. Different lowercase letters represent significant difference (*p* < 0.05) compared between groups.
